# Green-synthesized magnetic core-shell NiFe_2_O_4_@Ag nanoparticles enhance antibiotic efficacy against nosocomial pathogens

**DOI:** 10.1016/j.bbrep.2025.102121

**Published:** 2025-07-01

**Authors:** Abolfazl Barzegar, Somayyeh Ebrahimzadeh, Mehri Haeili, Yalda Shoghi, Alireza Ahmadi

**Affiliations:** a Department of Animal Biology, Faculty of Natural Sciences, University of Tabriz, Tabriz, Iran; b Molecular Medicine Research Center, Tabriz University of Medical Sciences, Tabriz, Iran; c Research Center of Biosciences and Biotechnology (RCBB), University of Tabriz, Tabriz, Iran

**Keywords:** Magnetic nanoparticles, NiFe_2_O_4_@Ag, Core-shell nanoparticles, Green nano-chemistry, Antibacterial activity, Nosocomial pathogens, Synergistic effects

## Abstract

Nosocomial infections pose a significant threat to patient health and healthcare systems globally. This study explores the synergistic potential of green-synthesized NiFe_2_O_4_@Ag core-shell nanoparticles in enhancing the efficacy of conventional antibiotics against multidrug-resistant nosocomial pathogens. The NiFe_2_O_4_@Ag nanoparticles were synthesized using a green method and characterized by X-ray diffraction (XRD), revealing particle sizes of 36.4 nm for NiFe_2_O_4_ and 51.54 nm for NiFe_2_O_4_@Ag. The antibacterial activity of these nanoparticles was evaluated alone and in combination with antibiotics—ciprofloxacin, tetracycline, and chloramphenicol—against five antibiotic-resistant bacterial strains associated with nosocomial infections, including Gram-negative *Escherichia coli*, *Pseudomonas aeruginosa*, *Acinetobacter baumannii*, and *Klebsiella pneumoniae*, as well as Gram-positive *Staphylococcus aureus*. NiFe_2_O_4_@Ag nanoparticles exhibited potent antimicrobial activity, with minimum inhibitory concentration (MIC) values of 256–512 mg/L). Notably, the combination of NiFe_2_O_4_@Ag nanoparticles with chloramphenicol and ciprofloxacin resulted in fractional inhibitory concentration index (FICI) values ranging from 0.25 to 0.75, indicating significant synergistic or additive effects, against most of tested gram-negative pathogens. While combination of NiFe_2_O_4_@Ag with ciprofloxacin exhibited the strongest enhancement, no synergistic effects were observed using NiFe_2_O_4_@Ag nanoparticles and tetracycline combinations for most tested pathogens (except for *E. coli*). The core-shell structure of NiFe_2_O_4_@Ag nanoparticles effectively integrates the magnetic properties of NiFe_2_O_4_ with the antimicrobial activity of silver, enabling potential magnetic separation and reducing environmental impact. Additionally, the nanoparticles exhibited low cytotoxicity in HFF-2 cells, suggesting good biocompatibility. These findings highlight the potential of NiFe_2_O_4_@Ag nanoparticles as a promising therapeutic strategy to combat multidrug-resistant nosocomial pathogens.

## Highlights

• Green synthesis of NiFe_2_O_4_@Ag NPs with enhanced antibacterial effectiveness.
• Synergistic with ciprofloxacin and chloramphenicol against Gram-negative strains.
• No synergistic interaction observed between NPs and tetracycline.
• NPs restore antibiotic activity against multidrug-resistant nosocomial pathogens.
• Low cytotoxicity and magnetic properties support safe biomedical applications.

## Graphical abstract

**Figure:**
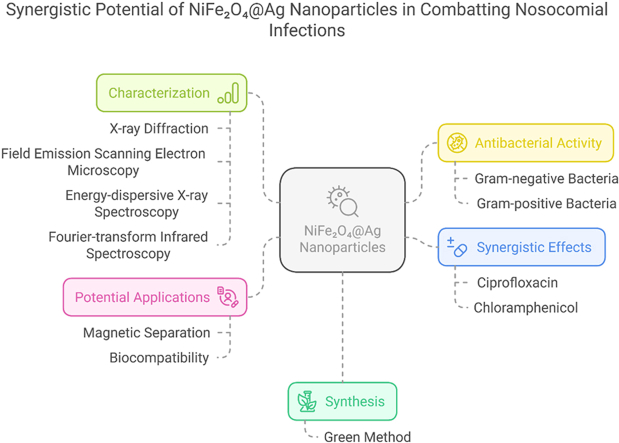

## 1 Introduction

Antimicrobial resistance (AMR) has emerged as one of the most critical global health challenges of the 21st century, with nosocomial or healthcare-associated infections being a significant contributor [1]. These infections, frequently caused by multidrug-resistant pathogens such as *Escherichia coli*, *Pseudomonas aeruginosa*, *Klebsiella pneumoniae*, *Acinetobacter baumannii*, and *Staphylococcus aureus*, which substantially compromise patient outcomes and strain healthcare systems worldwide [2,3]. The overuse and misuse of antibiotics in clinical settings have accelerated the evolution of resistant strains, rendering many conventional therapies ineffective [4]. Reports indicate that AMR is now implicated in nearly 60 % of all hospital-acquired infections, highlighting the urgent need for novel antimicrobial strategies [1].

A wide range of strategies has been investigated to mitigate AMR, including the discovery of novel antibiotics, the implementation of combination therapies, targeting resistance-associated enzymes or proteins, and the application of advanced physicochemical systems [5]. While these approaches show promise, they are often constrained by long development timelines, high costs, and diminishing effectiveness due to the rapid evolution of microbial resistance mechanisms. In recent years, nanotechnology—particularly the use of metallic nanoparticles—has emerged as a complementary or alternative strategy [6]. Among these, silver nanoparticles (AgNPs) have attracted considerable interest due to their potent broad-spectrum antibacterial properties and their capacity to disrupt biofilm formation [7]. However, challenges related to AgNP cytotoxicity and environmental persistence restrict their standalone application in biomedical settings. To overcome the limitations of AgNPs, core-shell nanosystems incorporating magnetic nanoparticles have emerged as a promising alternative. Core–shell hybrid nanoparticles are often fabricated using noble or functional materials such as Au, SiO_2_, or Ag [[8], [9], [10], [11]]. In these systems, a functional or inert shell stabilizes the magnetic core and provides a platform for surface modification [12]. When antimicrobial agents such as silver are integrated into the shell, the resulting nanostructures exhibit enhanced antibacterial efficacy, improved targeting of microbial biomolecules, and reduced toxicity and aggregation, thereby limiting the development of resistance [13].

Among different magnetic nanomaterials, nickel ferrite (NiFe_2_O_4_), a soft and smooth magnetic semiconductor, has attracted growing interest due to its catalytic possessions, chemical stability, cost-effectiveness, and multifunctionality [[14], [15], [16], [17]]. However, like other magnetic nanoparticles, NiFe_2_O_4_ tends to aggregate under physiological conditions and may set off cytotoxic outcomes, such as DNA damage [18,19]. To mitigate these issues, the present study proposes the green synthesis of magnetic core–shell NiFe_2_O_4_@Ag nanoparticles, which synergistically combine the magnetic properties of nickel ferrite and the antimicrobial capabilities of silver [20]. Previous reports have demonstrated the antimicrobial efficacy of green-synthesized NiFe_2_O_4_ nanoparticles, supporting the feasibility of this approach [[21], [22], [23]].

Consequently, this study aims to green-synthesize and characterize NiFe_2_O_4_@Ag core-shell nanoparticles and evaluate their antibacterial efficacy against multidrug-resistant common nosocomial pathogens. Furthermore, their synergistic activity with conventional antibiotics and cytocompatibility in HFF-2 cells are assessed to establish their potential as biocompatible antimicrobial agents.

## 2 Materials and methods

Ferric chloride (FeCl_3_·6H_2_O), nickel chloride (NiCl_2_·7H_2_O), silver nitrate (AgNO_3_), sodium hydroxide, ethanol (EtOH), and other chemicals were procured in analytical grade from Merck and used as acquired. Dimethyl sulfoxide (DMSO), MTT reagent, ciprofloxacin, tetracycline, and chloramphenicol powders were supplied via Sigma-Aldrich (St. Louis, MO, USA). Phosphate-buffered saline (PBS), RPMI 1640 medium, fetal bovine serum (FBS), Trypsin, and Penicillin/Streptomycin were purchased from Gibco (Invitrogen, NY, USA). The human foreskin fibroblast cell line (HFF-2) was purchased from the Pasteur Institute, Tehran, Iran. Deionized water was used for all experimental procedures to ensure consistency and accuracy. The *Arnebia euchroma* root used in this study was sourced from Darudarman Salafchegan (https://darudarmanco.com/en/about-us), which holds all associated collection data including location, methodology, and permits.

### 2.1 Green synthesis of core-shell NiFe_2_O_4_@Ag NPs

The spherical NiFe_2_O_4_ nanomaterials have been synthesized the use of a hydrothermal approach in an alkaline solution (pH > 10) with *Arnebia euchroma* extract as a green reducing and stabilizing agent. Separate aqueous solutions of NiCl_2_·6H_2_O (0.1 M, 20 mL) and FeCl_3_·6H_2_O (0.2 M, 20 mL) were prepared and blended thoroughly in a molar ratio of 1:2, leading to a final volume of 40 mL. For the green synthesis of NiFe_2_O_4_ NPs, 20 mL of ethanolic *A. euchroma* extract —rich in shikonin, a remarkable antioxidant and reducing agent [24]— was progressively added to the salt solution. Shikonin, a member of the anthraquinone family, is a key pharmaceutical compound with extensive reducing properties [24]. The mixture sample was stirred magnetically for 30 min, and pH was adjusted to 13 with 1 M NaOH. Subsequently, the suspension was transferred into a Teflon-coated autoclave and heated at 160 °C for 24 h, leading to the precipitation of darkish brown NiFe_2_O_4_ nanoparticles. The precipitate washed with ethanol (three times), separated using an external magnet, and dried in an oven at 70 °C for 24 h, yielding reddish-brown NiFe_2_O_4_ nanoparticles.

To construct core-shell NiFe_2_O_4_@Ag nanoparticles, silver deposition was accomplished through a hydrothermal approach. In this method, 1.0 g of NiFe_2_O_4_ nanoparticles (NPs) was dispersed in 20 mL of deionized water containing *A. euchroma* extract, accompanied via ultrasonic agitation for 30 min. A solution of AgNO_3_ (1 g/mL), dissolved in 15 mL of deionized water, was step by step introduced to the NiFe_2_O_4_ suspension in a concentration ratio of 1:1. Subsequently, the mixture was moved to autoclave and subjected to hydrothermal conditions at 200 °C for 4 h, enabling the silver deposition process.

### 2.2 Characterization of core-shell NiFe_2_O_4_@Ag NPs

The synthesized NiFe_2_O_4_@Ag NPs were comprehensively characterized to evaluate their structural, morphological, and chemical properties. X-ray diffraction (XRD) analysis was performed using a TD-3700 diffractometer (Shimadzu, Japan) operated with Cu Kα radiation (λ = 1.5406 Å) at 40 kV and 30 mA, covering a 2θ range of 5°–85° at room temperature. The average crystallite size was estimated using the Scherrer equation:

$$D=\frac{K\lambda }{\beta \;\cos\;\theta }$$

where *D* is the average crystallite size (nm), *K* is the Scherrer constant (0.9), *λ* is the X-ray wavelength, *β* is the full width at half maximum (FWHM) of the diffraction peaks, and *θ* is the Bragg angle. This method assumes that peak broadening arises solely from finite crystallite size and neglects strain effects [25]. Field emission scanning electron microscopy (FE-SEM) was carried out using a MIRA3 FEG-SEM system (Tescan, Czech Republic) to research the morphology and surface features of the synthesized nanoparticles. Elemental composition was determined by energy-dispersive X-ray (EDX) spectroscopy using a TESCAN VEGA3 SEM operated at 15 kV [26].

Fourier-transform infrared spectroscopy (FT-IR) was employed to identify the functional groups involved in nanoparticle stabilization. The measurements were conducted using a Bruker Tensor 27 spectrometer (Germany) over a spectral range of 4000–400 cm^−1^ with a resolution of 4 cm^−1^ [27] Samples were prepared using the KBr pellet method by mixing the sample with spectroscopic grade KBr at a 1:100 (w/w) ratio, followed by compression under 10 tons of pressure for 2 min.

Dynamic Light Scattering (DLS) analysis was carried out using a Zeta sizer Nano ZS (Malvern Instruments) at 25 °C to determine the hydrodynamic size distribution of the nanoparticles. All measurements were performed in aqueous suspension at pH 7.2, adjusted using phosphate-buffered saline (PBS).

### 2.3 Preparation of stock solution of NiFe_2_O_4_@Ag and antibiotics

Stock concentration of NiFe_2_O_4_@Ag in all experiments was 10 mg/mL in sterile deionized water which was ultrasonicated to improve particle dispersion. NPs were then serially diluted by twofold dilution in the range of the final concentration of 60–1000 μg/mL, then kept at room temperature until use. Tetracycline, chloramphenicol, and ciprofloxacin stock solutions were prepared at 10 mg/ml. Briefly, 50 μL of each sample of NPs and antibiotic concentration (total 100 μL of each antibiotic– of NiFe_2_O_4_@Ag combination) were transferred to each well of a 96-well plate after a range of concentrations of NiFe_2_O_4_@Ag (16–1024 μg/mL) and the antibiotics (0.07–256 μg/mL) were prepared by serial dilution.

### 2.4 Antibacterial susceptibility testing

We evaluated the antibacterial activity of NiFe_2_O_4_@Ag nanoparticles, alone and in combination with different antibiotics, against five clinical MDR bacterial isolates of hospital origin including 4 g-negative (*Escherichia coli*, *Pseudomonas aeruginosa*, *Acinetobacter baumannii*, and *Klebsiella pneumoniae*) and 1 g-positive (*Staphylococcus aureus*) bacteria. Conventional biochemical assays were used to identify the isolates to the species level [28]. We employed the agar well diffusion assay for qualitative analysis and the broth microdilution method for quantitative assessment.

#### 2.4.1 Agar well diffusion assay

The antibacterial activity of NiFe_2_O_4_ and NiFe_2_O_4_@Ag nanoparticles against five clinical multidrug-resistant (MDR) bacterial strains [29] was qualitatively assessed using the agar well diffusion method, which revealed significant zones of inhibition (ZOI), particularly for NiFe_2_O_4_@Ag nanoparticles. Overnight cultures of each bacterial strain were adjusted to a turbidity equivalent to a 0.5 McFarland standard (approximately 10^8^ CFU/mL) and spread onto Mueller-Hinton agar plates. Wells with a diameter of 5 mm and a depth of 4 mm were then punched into the agar, and each well was filled with 60 μL of a 10 mg/mL suspension of either NiFe_2_O_4_ or NiFe_2_O_4_@Ag nanoparticles. The nanoparticle suspensions were prepared by dissolving the nanoparticles in sterile water, followed by ultrasonication for 20 min to ensure uniform dispersion. A tigecycline disk (15 μg) served as a positive control. After incubating the plates at 37 °C for 24 h, the mean inhibition zones’ diameters were measured based on three replicates to determine the antibacterial activity of tested compounds.

#### 2.4.2 Broth microdilution method

For quantitative assessment, we determined the minimum inhibitory concentrations (MICs) and minimum bactericidal concentrations (MBCs) of NiFe_2_O_4_@Ag nanoparticles alone, and in combination with the antibiotics of ciprofloxacin, tetracycline, and chloramphenicol, collectively referred to as NiFe_2_O_4_@Ag-antibiotics. These methodologies facilitated a comprehensive evaluation of the antibacterial efficacy of NiFe_2_O_4_@Ag nanoparticles against multidrug-resistant nosocomial pathogens when utilized in synergy with common human antibiotics. Overnight bacterial cultures were diluted to achieve a turbidity conforming to the 0.5 McFarland standard (approximately 10^8^ CFU/mL). These suspensions were subsequently inoculated into Mueller-Hinton broth containing serial dilutions of NiFe_2_O_4_@Ag nanoparticles (16–1024 μg/mL) or the antibiotics (0.007–256 μg/mL) yielding a final concentration of 5 × 10^5^ CFU/mL of tested bacteria. The broth microdilution method was performed in triplicate, according to standard protocols. Following an incubation period at 35–37 °C for 16–20 h, the MIC was determined as the lowest concentration of agent that successfully inhibited visible bacterial growth. The MBC was identified as the lowest concentration of agent that resulted in no bacterial growth on the agar plates.

### 2.5 Synergistic antibacterial testing of NiFe_2_O_4_@Ag–antibiotics

To evaluate the synergistic antibacterial effects of NiFe_2_O_4_@Ag nanoparticles (NPs) combined with antibiotics, we calculated the fractional inhibitory concentration index (FICI) following the guidelines provided by the European Committee on Antimicrobial Susceptibility Testing (EUCAST) [30]. The FICI was determined using the following formulas:

$${\text{FIC}}_{NPs}=\frac{{\text{MIC}}_{NPs-ATB}\;}{{\text{MIC}}_{NPs}}$$

$${\text{FIC}}_{ATB}=\frac{{\text{MIC}}_{ATB-NPs}}{{\text{MIC}}_{ATB}}$$

$$\text{FICI}={\text{FIC}}_{NPs}+{\text{FIC}}_{ATB}$$

Where FIC_NPs_ represents the FIC of NiFe_2_O_4_@Ag NPs, FIC_ATB_ denotes the FIC of the applied antibiotics, and MIC_NPs-ATB_ denotes the MIC of combined NiFe_2_O_4_@Ag-antibiotics. FICI is commonly used to define the synergistic effects of agents for the inhibition of bacteria. An average FICI was calculated in order to classify the combined antibacterial effect of antibiotics and NiFe_2_O_4_@Ag NPs as synergistic (FICI ≤0.5), additive (0.5 < FICI ≤1), indifferent (1< FICI <4), and antagonistic (FICI ≥4). These classifications are commonly used to define the interactions between NPs and common human antibiotics for bacterial growth inhibition.

### 2.6 Cellular cytotoxicity of NiFe_2_O_4_@Ag NPs

The cytotoxic outcomes of NiFe_2_O_4_@Ag NPs have been evaluated using the Human Foreskin Fibroblast (HFF-2) cell line. HFF-2 cells were well cultured in the complementary RPMI1640 medium with 10 % embryonal serum (FBS; GIBCO Invitrogen) and 1 % penicillin/streptomycin at 37° and 5 % CO_2_. Cells had been sub-cultured every 2–3 days to keep ideal growth and viability, with the culture medium replaced daily until the cells are ready to be tested. The thiazolyl blue tetrazolium (MTT) assay was performed for cytotoxicity assessment, which measures mitochondrial metabolic activity as an indicator of cellular viability [31]. HFF-2 cells were seeded into 96-well plates at a density of 1 × 10^5^ cells/well and allowed to adhere. Subsequently, NiFe_2_O_4_ and NiFe_2_O_4_@Ag nanoparticles have been introduced at concentrations up to 500 μg/mL for 24, 48 and 72 h incubation at 37 °C. After the incubation time, 20 μL of MTT solution in PBS was added into each well, and the plates were incubated for a further 3 h at 37 °C to allow the formation of formazan crystals inside metabolically active cells. Following the elimination of the medium, 100 μL of dimethyl sulfoxide (DMSO) was added to each well to dissolve the formazan crystals. The plates had been shaken for 20 min to ensure complete dissolution. The absorbance changes at 570 nm were measured using a DANA-3200 Elisa reader to evaluate the possible cytotoxic properties of NiFe_2_O_4_ and NiFe_2_O_4_@Ag nanoparticles with data analysis by GraphPad Prism 10 software program. This approach provides quantitative insights for the possibly cytotoxic effects of nanoparticles, making sure a comprehensive evaluation of their biocompatibility for therapeutic applications.

### 2.7 Statistical analysis

In this work, we evaluated the cytotoxic effects of NiFe_2_O_4_ and NiFe_2_O_4_@Ag nanoparticles using statistical methods. The *t*-test was used to assess differences between two particular groups. P < 0.05, or a p-value below 0.05, was regarded as statistically significant.

## 3 Results

### 3.1 XRD analysis of NiFe_2_O_4_ and NiFe_2_O_4_@Ag nanoparticles

**Fig. 1:**
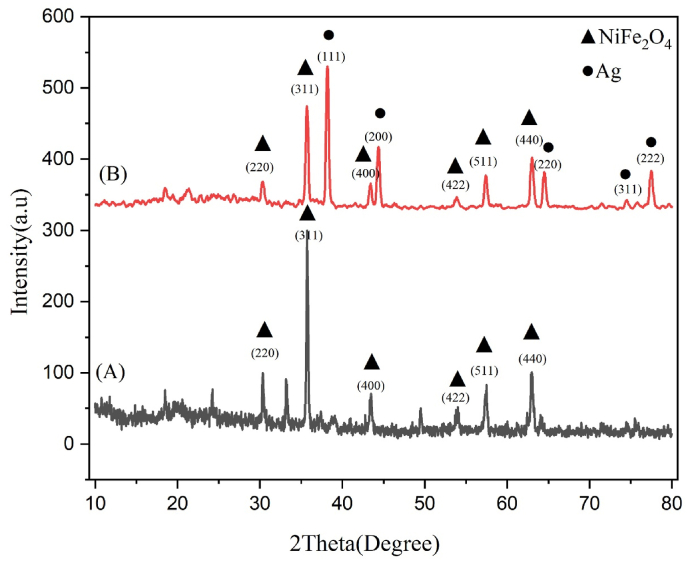
XRD patterns of the synthesized NiFe_2_O_4_ nanoparticles (A) and NiFe_2_O_4_@Ag core-shell nanoparticles (B), showcasing the cubic spinel structure of NiFe_2_O_4_ and the distinct diffraction peaks corresponding to metallic silver in NiFe_2_O_4_@Ag.

The cubic spinel structure was reflected in the XRD pattern of NiFe_2_O_4_ nanoparticles (Fig. 1). According to Bragg's rule, these reflections contained typical characteristic peaks (220), (311), (400), (422), (511), and (440). The produced nickel ferrite's nanocrystalline nature was demonstrated by the strong peaks. All observed reflections matched the expected fcc-type cubic spinel structure, with no additional peaks detected, confirming the absence of impurities. Following additional validation of the single-phase cubic spinel structure of NiFe_2_O_4_, the average crystallite size was determined to be 36.4 nm, which is in accordance with JCPDS card No. 01-074-2081.

For NiFe_2_O_4_@Ag nanoparticles, the XRD pattern additionally exhibited reflections attributed to metallic silver. Distinct diffraction peaks were observed at 2θ values of 38.2°, 44.4°, 64.5°, 77.5°, and 81.6°, corresponding to the (111), (200), (220), (311), and (222) planes, respectively, and aligned with the JCPDS File No. 01-087-0597. These strong peaks indicated the high crystallinity of the deposited silver layer on the NiFe_2_O_4_ core. Due to the inclusion of the silver shell coating, the crystallite size of the NiFe_2_O_4_@Ag nanoparticles was found to be 51.54 nm, which is larger than the 36.4 nm for NiFe_2_O_4_. The outcomes demonstrated the efficacy of the green synthesis technique by validating the successful synthesis of high-purity NiFe_2_O_4_ and NiFe_2_O_4_@Ag nanoparticles with distinct crystalline structures.

### 3.2 Morphology of NiFe_2_O_4_ and NiFe_2_O_4_@Ag nanoparticles

**Fig. 2:**
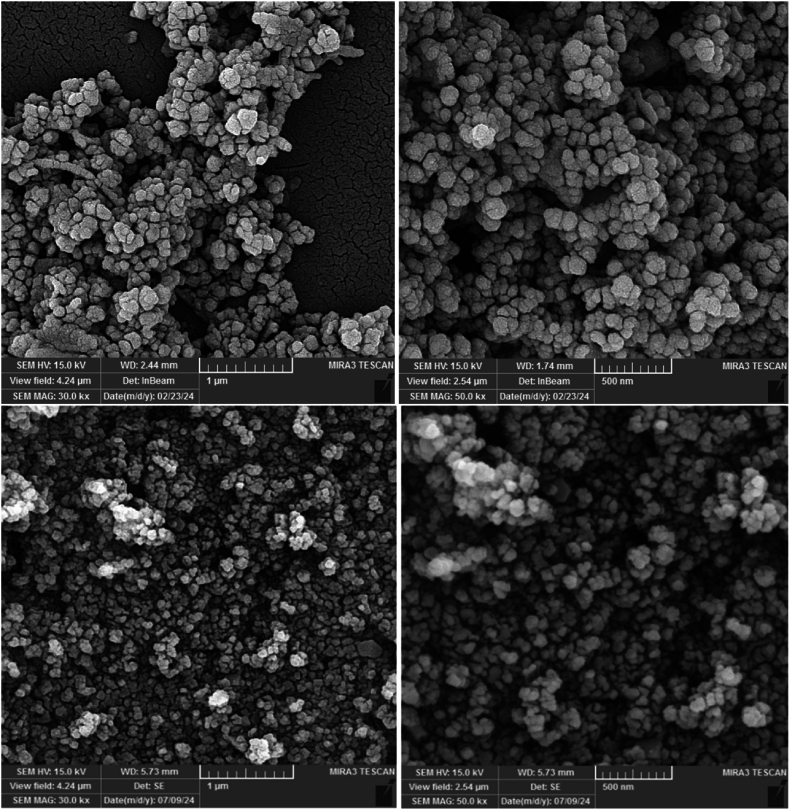
SEM images of synthesized NiFe_2_O_4_ nanoparticles (upper panels) and NiFe_2_O_4_@Ag core-shell nanoparticles (lower panels).

**Fig. 3:**
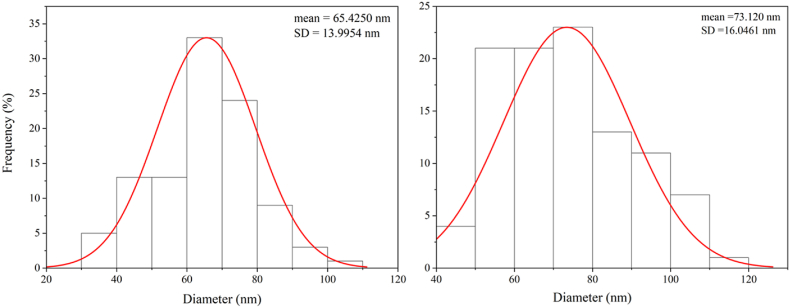
Particle size distribution of synthesized (A) NiFe_2_O_4_ and (B) NiFe_2_O_4_@Ag. Particle size was determined by measuring approximately 150 nanoparticles from three randomly selected high-magnification SEM images using ImageJ software.

Fig. 2, Fig. 3 display the SEM images related to nanoparticle size distributions. NiFe_2_O_4_ nanoparticles' propensity for micrometer-scale particle aggregation was discovered by SEM examination. The propensity of NiFe_2_O_4_ nanoparticles to cluster due to their high surface energy promotes aggregation into larger assemblies. The average size of individual NiFe_2_O_4_ nanoparticles was found to be roughly 65 nm in spite of this aggregation. In addition, Fig. 3 showed that the NiFe_2_O_4_@Ag nanoparticles had a spherical shape and a rather homogeneous size distribution. The average size of the NiFe_2_O_4_@Ag nanoparticles was found to be 73 nm, which is slightly larger than the NiFe_2_O_4_ core particles due to the silver shell covering. The green synthesized NiFe_2_O_4_@Ag NPs, similar to pyridine-2,3-dicarboxylate-based metal–organic frameworks [32], generated a constant core-shell structure, as confirmed by SEM showing particle size distribution and morphology. Therefore, appropriate surface modification and stabilization are crucial to prevent aggregation and enhance the usage of magnetic nanoparticles in a range of therapeutic domains.

### 3.3 EDX and elemental mapping of NiFe_2_O_4_ and NiFe_2_O_4_@Ag NPs

**Fig. 4:**
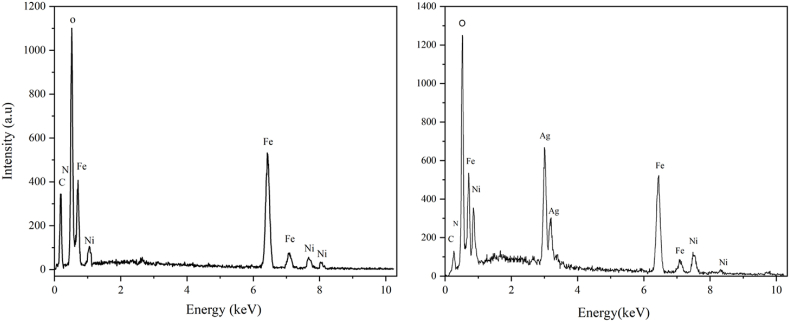
EDX spectrum of NiFe_2_O_4_ (left panel) and NiFe_2_O_4_@Ag (right panel) nanoparticles, confirming the presence of Fe, O, Ni, and Ag, along with carbon and oxygen, which are attributed to the Arnebia euchroma extract used in the green synthesis process.

The EDX spectrum (Fig. 4) confirmed the presence of Fe, O, and Ni, consistent with the formation of NiFe_2_O_4_ nanoparticles. In the case of NiFe_2_O_4_@Ag nanoparticles, the spectrum additionally confirmed the presence of Ag, further validating the successful synthesis of the core-shell structure. The presence of carbon in the spectra can be attributed to the use of *A. euchroma* extract as a reducing and stabilizing agent during the green synthesis process. Importantly, no peaks corresponding to impurities were observed, underscoring the high purity of the synthesized nanoparticles.

**Fig. 5:**
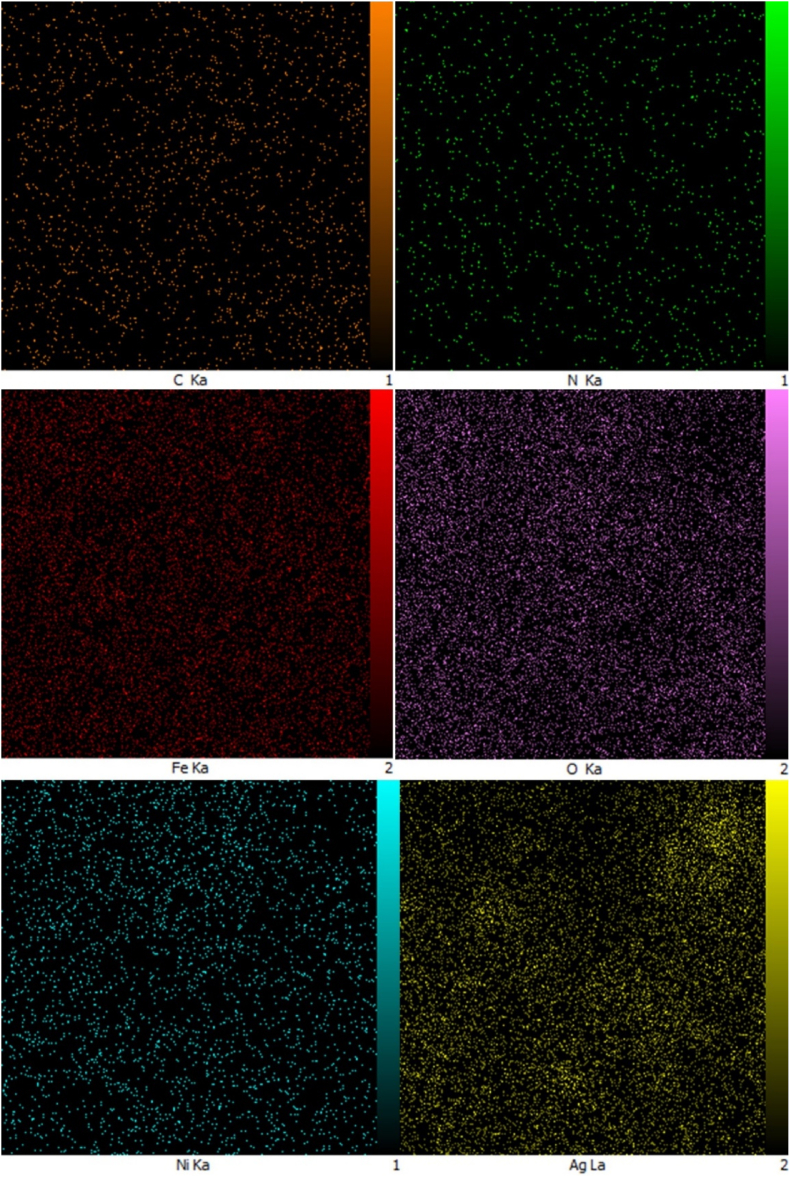
Elemental mapping analysis for NiFe_2_O_4_@Ag nanoparticles, illustrating the homogeneity of the distributions of C, N, Fe, O, Ni, and Ag atoms.

Elemental mapping in Fig. 5 further demonstrated a uniform distribution of Ni, Fe, and O within the NiFe_2_O_4_ core, and a homogeneous dispersion of Ag on the nanoparticle surface. The presence of carbon and nitrogen elements also confirmed the incorporation of *A. euchroma* extract components, validating its role in the biosynthesis and capping of the nanoparticles.

### 3.4 FT-IR analysis of NiFe_2_O_4_ and NiFe_2_O_4_@Ag NPs

**Fig. 6:**
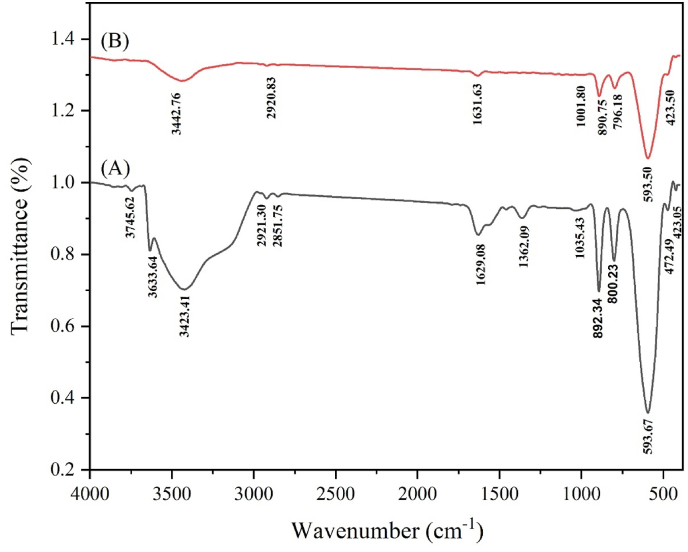
FT-IR spectra of NiFe_2_O_4_ (A) and NiFe_2_O_4_@Ag (B) nanoparticles, showing characteristic metal-oxygen bands and functional group vibrations associated with the *Arnebia euchroma*-mediated synthesis process.

For NiFe_2_O_4_ nanoparticles, the spectrum displayed characteristic metal-oxygen bands at 673 cm^−1^ and 554 cm^−1^, corresponding to ion vibrations in the cubic spinel crystal lattice (Fig. 6). A broad band at 3403 cm^−1^ was observed, which is associated with the symmetric vibration of hydroxyl (-OH) groups. Additionally, a peak at 1362 cm^−1^ indicated the CH bending region, with another notable peak at 1317 cm^−1^ ascribed to the distinctive bending of methyl (-CH_3_) groups.

The FT-IR spectrum of NiFe_2_O_4_@Ag nanoparticles (Fig. 6) exhibited additional bands compared to the NiFe_2_O_4_ spectrum, highlighting the modifications caused by silver deposition and the incorporation of biomolecules from the green synthesis process. The band at 1629 cm^−1^, attributed to carbonyl (C=O) stretching vibrations in amide I bonds, suggests the involvement of biocompounds in the capping or stabilization of the nanoparticles. The spectrum retained the characteristic metal-oxygen bands of NiFe_2_O_4_, confirming that the ferrite core structure was preserved during the synthesis of NiFe_2_O_4_@Ag nanoparticles.

Overall, the FT-IR analysis confirmed the successful functionalization of the NiFe_2_O_4_@Ag nanoparticles and highlighted the role of *A. euchroma* extract in their green synthesis, as evidenced by characteristic biomolecular groups such as hydroxyl, amines, and esters. These findings align with previous characterization of green synthesized phenyl benzofuran derivatives via C–H functionalization to C–O and C–C bond formation [33].

### 3.5 Antibacterial activity of NiFe_2_O_4_@Ag NPs combined with antibiotics

In the results analysis, NiFe_2_O_4_@Ag nanoparticles exhibited notable antibacterial efficacy, as evidenced by the agar well diffusion assay. As shown in Table 1, they produced clear zones of inhibition against all five clinical MDR bacterial strains, including both Gram-negative and Gram-positive bacteria, underscoring their potential as effective antimicrobial agents.

**Table 1:** Antibacterial activity of NiFe_2_O_4_ and NiFe_2_O_4_@Ag nanoparticles against five clinical MDR bacteria determined by agar well diffusion assay.

Sample: Gram ±	Bacterial Strain	ZOI (mm)^a^	
		NiFe_2_O_4_	NiFe_2_O_4_@Ag
Gram-negative	*E. coli*	–	7.0
Gram-negative	*P. aeruginosa*	–	11.0
Gram-negative	*A. baumannii*	–	10.0
Gram-negative	*K. pneumoniae*	–	–
Gram-positive	*S. aureus*	–	9.0

a ZOI:zones of inhibition.

Additionally, the minimum inhibitory concentration (MIC) and minimum bactericidal concentration (MBC) of NiFe_2_O_4_@Ag NPs, individual antibiotics, and their combinations (NiFe_2_O_4_@Ag-antibiotics) were determined, with results summarized in Table 2. Notably, the combination treatments showed significant synergistic effects, particularly against key Gram-negative bacteria. For instance, NiFe_2_O_4_@Ag NPs increased sensitivity of *E. coli*, *A. baumannii*, and *K. pneumoniae* to chloramphenicol and *E. coli*, *A. baumannii*, *P. aeruginosa*, and *K. pneumoniae* to ciprofloxacin, thereby strengthening the antibacterial activity of these antibiotics. Moreover, as presented in Table 3, the combination of chloramphenicol with NiFe_2_O_4_@Ag NPs featured FICI values of 0.258 for *E. coli* and 0.50 for *K. pneumoniae* (indicating full synergism). All combinations with ciprofloxacin showed effectiveness/additive effect, with FICI values ranging from 0.25 to 0.75 for all tested gram-negative bacteria. However, no positive effects were observed for tetracycline against any bacterial strains except for *E. coli* where a synergistic interaction was observed (FICI = 0.187).

**Table 2:** MIC and MBC values of NiFe_2_O_4_@Ag and clinically important antibiotics (mg/l).

Strain	NPs		Cip		Cip-NP		Tet		Tet-NPs		Chl		Chl-NPs	
	MIC	MBC	MIC	MBC	MIC	MBC	MIC	MBC	MIC	MBC	MIC	MBC	MIC	MBC
*E. coli*	256	>512	128	128	64	128	128	256	16	256	8	64	2	32
*A. baumannii*	256	512	256	>256	32	32	32	64	32	64	256	>256	128	>256
*P. aeruginosa*	256	>512	128	128	64	128	32	64	32	128	256	>256	256	256
*K. pneumoniae*	512	>512	128	>128	32	>128	16	>256	16	256	>256	>256	128	>128
*S. aureus*	512	512	8	8	8	8	128	>256	128	>256	128	>128	128	>128

*Abbreviations:* NPs; NiFe_2_O_4_@Ag nanoparticles, Chl; chloramphenicol, Cip; ciprofloxacin, Tet; tetracycline, MBC; minimum bactericidal concentration, MIC; minimum inhibitory concentration.

**Table 3:** Average FIC and FICI values representing the effect of NiFe_2_O_4_@Ag nanoparticles in combination with conventional antibiotics.

Strain	Cip		Cip-NP	Tet		Tet-NPs	Chl		Chl-NPs
	FIC_NPs	FIC	FICI	FIC_NPs	FIC	FICI	FIC_NPs	FIC	FICI
*E. coli*	0.25	0.50	**0.75**	0.0625	0.125	**0.187**	0.0078	0.25	**0.258**
*A. baumannii*	0.125	0.125	**0.25**	0.125	1.0	**1.125**	0.50	0.50	**1.0**
*P. aeruginosa*	0.25	0.5	**0.75**	0.125	1.0	**1.125**	1.0	1.0	**2.0**
*K. pneumoniae*	0.0625	0.25	**0.313**	0.0312	1.0	**1.0312**	0.25	0.5	**≤0. 50**
*S. aureus*	0.01562	1.0	**1.016**	0.25	1.0	**1.25**	0.25	1.0	**1.25**

*Abbreviations:* NPs; NiFe_2_O_4_@Ag nanoparticles, Cip; ciprofloxacin, Tet; tetracycline, Chl; chloramphenicol, FIC; fractional inhibitory concentration values, FICI; fractional inhibitory concentration index values, FIC_NPs; FIC values of nanoparticles in combination with related antibiotic. Synergistic and additive values are highlighted with underlines.

While NiFe_2_O_4_@Ag NPs demonstrated intrinsic antibacterial activity against *S. aureus*, their combination with antibiotics did not yield enhanced efficacy against this pathogen. This lack of synergism may be attributed to specific interaction dynamics between the nanoparticles, antibiotics, and the bacterial cell wall structure or other specific targets of *S. aureus*.

### 3.6 Cytotoxic effect of NiFe_2_O_4_@Ag

**Fig. 7:**
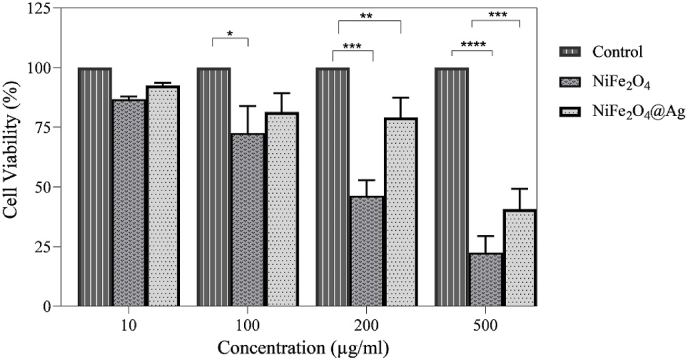
Cytotoxicity of synthesized NiFe_2_O_4_ and NiFe_2_O_4_@Ag in HFF-2 human cells based on the MTT approach. ∗, ∗∗, ∗∗∗, and ∗∗∗∗ represent p-value <0.05, 0.01. 0.001, and 0.0001, respectively.

The cytotoxic effects of NiFe_2_O_4_ and NiFe_2_O_4_@Ag NPs were presented in Fig. 7 using the MTT assay on HFF-2 cells, a normal cell line, over a 48-h exposure period. The results revealed that cell viability decreased progressively with increasing concentrations of NiFe_2_O_4_ NPs, up to 200 μg/mL. However, NiFe_2_O_4_@Ag NPs demonstrated no significant cytotoxicity at the same concentration, indicating reduced toxicity compared to NiFe_2_O_4_ NPs. The observed cytotoxicity of NiFe_2_O_4_@Ag NPs is consistent with previous reports. For instance, earlier studies indicated that NiFe_2_O_4_@Ag NPs significantly increased apoptotic cell frequency in adenocarcinoma gastric cell lines, while exhibiting lower cytotoxic effects on normal cell lines compared to cancer cell lines [34]. Moreover, other investigations have demonstrated that Ni/NiFe_2_O_4_–GO nanocomposites can induce oxidative stress, ultimately leading to bacterial cell death in *E. coli* [35]. These findings suggest that while NiFe_2_O_4_@Ag NPs exhibit potent antibacterial properties, their relatively lower cytotoxicity at higher concentrations makes them a promising candidate for biomedical applications, though careful consideration of dosing is necessary. Consequently, appropriate surface modification enhances the biocompatibility, stability, and functionality of magnetic NPs, making them more suitable for biomedical applications.

## 4 Discussion

Characterization techniques were used to confirm that NiFe_2_O_4_@Ag nanoparticles were produced effectively. X-ray diffraction examination demonstrated the successful formation of both NiFe_2_O_4_ and NiFe_2_O_4_@Ag nanoparticles, as well as the characteristic cubic spinel structure of NiFe_2_O_4_ and the characteristic peaks of metallic silver in the core-shell structure. The synthesized NiFe_2_O_4_@Ag NPs, similar to MgFe_2_O_4_@SiO_2_@CBCE-Pd [25], yielded a stable core-shell structure, as confirmed by XRD analyses showing spinel structure.

The form and size distribution of the nanoparticles were shown by FE-SEM imaging, demonstrating that the NiFe_2_O_4_ core had been successfully coated with silver. On the NiFe_2_O_4_ core, the presence and uniform distribution of silver were further validated by elemental mapping analysis and EDX. The combined EDX and elemental mapping results provide strong evidence for the successful synthesis of high-purity NiFe_2_O_4_ and NiFe_2_O_4_@Ag nanoparticles with uniform elemental distribution, facilitated by the *A. euchroma* extract. Consistent with the characterization of Fe_3_O_4_@SiO_2_ core-shell NPs [27], EDX analysis of NiFe_2_O_4_@Ag verified Ag coating. These findings ensure the structural and functional reliability of the nanoparticles for potential biomedical applications.

The silver shell provides antibacterial activity and reduces potential toxicity while the magnetic NiFe_2_O_4_ core allows for potential magnetic separation, and long-term exposure. The presence of carbon in the EDX spectra further supported the hypothesis that *A. euchroma* extract was utilized as a stabilizing and reducing agent during the green synthesis process, according to FT-IR studies. Our green synthesis of NiFe_2_O_4_@Ag NPs, using *A. euchroma* extract, aligns with eco-friendly approaches reported for MgFe_2_O_4_@SiO_2_@CBCE-Pd [12] and Fe_3_O_4_-based catalysts [25,27], ensuring scalability and environmental safety. In addition, the result of synthesized NiFe_2_O_4_@Ag core-shell characterization was aligned with recent studies [12,25,27], validating successful synthesis and stability of the NPs. Catalytic nanomaterials have diverse applications, from fluorescence detection [36] to antimicrobial enhancement, as demonstrated by our NiFe_2_O_4_@Ag NPs’ interaction with pathogens. Moreover, the catalytic activity of NiFe_2_O_4_@Ag NPs, which enhances antibiotic efficacy, aligns with the electrocatalytic properties of Co(II)-MOFs [26], indicating the potential of metal-based nanomaterials in biomedical applications. Also, the catalytic enhancement of antibiotic efficacy by NiFe_2_O_4_@Ag NPs may involve heterogeneous catalysis, similar to coordinated polymers used in Henry reactions [37], highlighting their surface-mediated activity. Nanostructured catalysts, such as Si_60_ nanocages for N_2_ reduction [38], demonstrate the versatility of nanomaterials, which we extend to antimicrobial applications with NiFe_2_O_4_@Ag NPs. The interaction of NiFe_2_O_4_@Ag NPs with pathogens may involve adsorption mechanisms, similar to those reported for 2D carbon nitride in mercury capture [39].

One of the key findings of the study is that NiFe_2_O_4_@Ag NPs had enhanced antibacterial activity against both Gram-positive and Gram-negative bacteria (Table 1). This is in line with previous research demonstrated that the ternary nanocomposite of NiFe_2_O_4_@HAp-Ag is more effective against some bacteria [40]. Notably, NiFe_2_O_4_@Ag NPs worked with conventional antibiotics to produce synergistic effects (Table 2, Table 3). A number of Gram-negative organisms, including *E. coli, A. baumannii, and K. pneumoniae,* revealed increased susceptibility to antibiotics such as ciprofloxacin and chloramphenicol by the NiFe_2_O_4_@Ag NPs, which improved their effectiveness against the tested pathogens. In a recent study, AgNPs were reported to significantly enhance the bactericidal activity of conventional antibiotics such as cefotaxime, ceftazidime, meropenem, ciprofloxacin, and gentamicin, against multidrug-resistant *Enterobacteriaceae* [7]. NiFe_2_O_4_@ABS@Ag nanocomposites exhibited significant antimicrobial properties, attributed to the composite materials [41]. Similarly, NiFe_2_O_4_@PANI@Ag nanocomposites showed potent antibacterial activity against both Gram-positive and Gram-negative bacteria. Notably, NiFe_2_O_4_@PANI@Ag nanocomposites outperformed several standard antibacterial drugs, including cefixime, penicillin, streptomycin, erythromycin, and amoxicillin, against Gram-negative *P. aeruginosa* [42]. Our findings denote that the synergistic effects of NiFe_2_O_4_@Ag nanoparticles combined with antibiotics are influenced by both the “bacterial species” and the “type of antibiotic” used. These findings align with previous studies demonstrating silver's ability to sensitize Gram-negative bacteria to vancomycin, thereby enhancing the antibacterial activity of this Gram-positive–specific antibiotic drug [43]. Compared to the previously reported systems in Table 4, the present study uniquely integrates a green synthesis approach with broad-spectrum antibacterial efficacy, demonstrating synergistic enhancement of multiple conventional antibiotics against clinically relevant MDR pathogens, thus offering a sustainable and highly effective antimicrobial strategy. Therefore, data highlight the potential of NiFe_2_O_4_@Ag NPs in potentiating antibiotic activity, providing a promising strategy for combating antibiotic-resistant bacterial infections. The combination of such nanomaterials with existing antibiotics could pave the way for innovative solutions in antimicrobial therapy.

**Table 4:** Comparative studies of synthesized nanoparticles with various structures, synthesis methods, and functional applications.

Material	Synthesis method	Type	Application	Performance	Ref.
NiFe_2_O_4_@Ag	Green Synthesis	Magnetic core-shell	Antimicrobial enhancement against MDR bacteria	Affecting both Gram-positive and negative	This work
NiFe_2_O_4_@PANI@Ag	Chemical reduction	Magnetic	Antimicrobial enhancement	Effecting both Gram-positive and negative	[42]
NiFe_2_O_4_@ABS@Ag	Chemical	Magnetic	Antimicrobial enhancement	Effecting both gram positive and negative	[41]
Fe_3_O_4_@DAA-Schiff-base/Pd	Chemical modification	Super Magnetic	C–N, C–C coupling yield	High yield	[27]
Pyridine-2,3-dicarboxylate metal-organic frameworks	Solvothermal	Non-Magnetic	Knoevenagel condensation yield/Organic synthesis	High catalytic efficiency	[32]
MgFe2O4@SiO2@CBCE-Pd	Green Synthesis	Magnetic core-shell	C–O and C–C cross-coupling reactions	High catalytic yield	[25]

The NPs work by multiple mechanisms of action which include rupturing bacterial cell membranes, interfering with DNA replication, and producing reactive oxygen species (ROS) [44]. The capacity of silver nanoparticles to emit Ag^+^ ions, which can damage bacterial cell membranes and obstruct essential cellular functions, is the main reason for their well-known broad-spectrum antibacterial qualities. The incorporation of Ag nanoparticles onto the NiFe_2_O_4_ core not only imparts magnetic properties but also enhances the dispersion and stability of the silver nanoparticles, leading to improved antibacterial performance. These mechanisms, combined with the action of the antibiotics, create a multi-pronged attack on the bacterial cell, making it more difficult for resistance development.

The MTT assay demonstrated the relatively low cytotoxicity of NiFe_2_O_4_@Ag nanoparticles towards HFF-2 cells, suggesting good biocompatibility. Previous studies indicated that NiFe_2_O_4_@Ag NPs significantly increased apoptotic cell frequency in adenocarcinoma gastric cell lines, while exhibiting lower cytotoxic effects on normal cell lines compared to cancer cell lines [34]. Moreover, other investigations have demonstrated that Ni/NiFe_2_O_4_–GO nanocomposites can induce oxidative stress, ultimately leading to bacterial cell death in *E. coli* [35]. These findings suggest that while NiFe_2_O_4_@Ag NPs exhibit potent antibacterial properties, their relatively lower cytotoxicity at higher concentrations makes them a promising candidate for biomedical applications, though careful consideration of dosing is necessary. Consequently, appropriate surface modification enhances the biocompatibility, stability, and functionality of magnetic NPs, making them more suitable for biomedical applications. Our cytotoxicity results align with the demonstrated that HFF-2 cell line viability remains unaffected by amino acid-coated iron oxide magnetic NPs, with highly biocompatible and non-toxic [45]. However, careful consideration of dosage and targeted delivery strategies will be essential for in vivo applications.

In conclusion, this study demonstrates the successful green synthesis of NiFe_2_O_4_@Ag core-shell nanoparticles with enhanced antibacterial activity and synergistic effects with conventional antibiotics. These findings highlight the promise of NiFe_2_O_4_@Ag core-shell nanoparticles as a potential therapeutic strategy to combat AMR, offering a new avenue for developing next-generation antimicrobial agents. Future research will focus on investigating the specific mechanisms of synergy, exploring targeted delivery strategies, and evaluating the in vivo efficacy and toxicity of these nanoparticles.

## 5 Future perspectives

These findings provide a solid foundation for advancing green-synthesized NiFe_2_O_4_@Ag core–shell nanoparticles as next-generation antimicrobial agents. Future research should prioritize in vivo evaluation of their antibacterial efficacy, biodistribution, and biosafety in appropriate animal models to validate their clinical potential. In particular, mechanistic investigations are needed to elucidate the molecular pathways underlying the observed synergistic effects with antibiotics such as ciprofloxacin and chloramphenicol, as well as to clarify the absence of synergy with tetracycline. Moreover, exploring the nanoparticles' effectiveness against bacterial biofilms—especially prevalent in nosocomial infections—could significantly enhance their therapeutic relevance. Given their magnetic properties, NiFe_2_O_4_@Ag NPs also present exciting opportunities for targeted drug delivery and magnetic hyperthermia, which could further improve therapeutic selectivity while minimizing systemic toxicity. Development of smart biomedical platforms, such as nanoparticle-embedded wound dressings, antimicrobial coatings for medical devices, or controlled-release formulations, could facilitate their clinical translation. Additionally, assessing the scalability and reproducibility of the green synthesis method will be crucial for enabling large-scale production. Collectively, these avenues represent critical next steps for translating NiFe_2_O_4_@Ag nanoparticles from bench to bedside in the fight against multidrug-resistant bacterial infections.

## 6 Conclusion

This study introduces a green synthesis method for NiFe_2_O_4_@Ag core-shell nanoparticles, which exhibit enhanced antibacterial activity against both Gram-positive and Gram-negative bacteria. NiFe_2_O_4_@Ag core-shell nanoparticles successfully sensitize a range of Gram-negative organisms to drugs like ciprofloxacin and chloramphenicol when used in conjunction with traditional antibiotics. The antibacterial qualities of silver and the magnetic capabilities of NiFe_2_O_4_ are successfully combined in the core-shell structure of NiFe_2_O_4_@Ag nanoparticles. The comparatively low cytotoxicity of HFF-2 cells increases their potential use in biomedicine. These results demonstrate the potential therapeutic application of NiFe_2_O_4_@Ag core-shell NPs in the fight against antibiotic resistance and provide a new approach for the creation of next-generation antimicrobial drugs.

## Availability of data and materials

The data and/or analysis from this study can be obtained from the corresponding author upon a reasonable request.

## Funding information

This research did not receive any specific grant from funding agencies in the public, commercial, or not-for-profit sectors.

## Declaration of competing interest

The authors declare that they have no known competing financial interests or personal relationships that could have appeared to influence the work reported in this paper.

## Data Availability

Data will be made available on request.
